# Stakeholders’ perspectives on facilitative supervision implementation in north-western Ghana: an exploration of adherence and quality of primary healthcare

**DOI:** 10.1017/S1463423622000457

**Published:** 2022-09-15

**Authors:** Faustina Sarkpoh, Maximillian Kolbe Domapielle

**Affiliations:** Department of Governance and Development Management, Faculty of Public Policy and Governance, Simon Diedong Dombo University of Business and Integrated Development Studies, P.O. Box UPW3, Wa, U.W.R, Ghana

**Keywords:** Facilitative supervision, primary healthcare, north-western Ghana

## Abstract

**Aim:**

Following growing concern about healthcare quality in many developing countries, this article analyses the relationship between facilitative supervision (FS) and the quality of primary healthcare (PHC) services in north-western Ghana.

**Background:**

While adherence to the tenets of FS aims to trigger improvement in the quality of PHC services, research has seldom explored this relationship to facilitate effective planning and implementation of PHC services, particularly in deprived areas.

**Methodology:**

Based on the implementation of FS in primary health facilities in a district and a municipality in north-western Ghana, a multi-case study approach was employed to collect and analyse the data. Specifically, 52 semi-structured interviews were conducted in the two study settings and the data were analysed using a thematic framework. Observation and secondary analysis were also employed to generate data to triangulate and supplement the interview data.

**Findings:**

The results reveal that health facilities in the Wa West district are relatively under-resourced, and this impedes the regularity of supervisory visits compared to the Wa Municipality. This notwithstanding, adherence to the prescriptions of FS is rated by the study participants as moderately satisfactory in both districts, culminating in improvement in the quality of PHC. This finding has implications for innovation in the mobilisation of health resources to increase the regularity of facilitation supervision in deprived settings. We advocate further research to establish whether the marginal improvement in the quality of PHC achieved in the two districts has resulted in an increase in uptake of PHC services to improve the health of the population or not.

## Introduction

**Figure 1.**
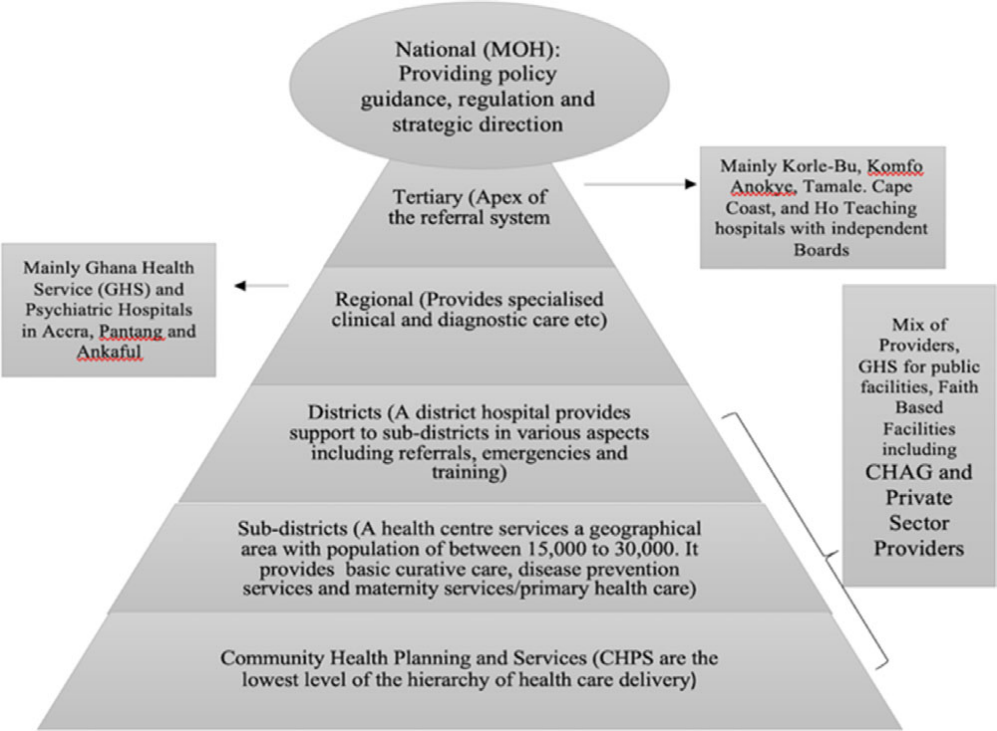
Structure of the health delivery system in Ghana

In the context of public health services provision, facilitative supervision (FS) is a refined form of participatory monitoring and evaluation (PM&E) involving self-introspection, reflexivity, and mutual learning from both the supervisor and the supervisee. As a key component of the Community Health Planning Services (CHPSs) programme, scholars and health practitioners view the implementation of FS as an important component of the broader strategy for achieving the objectives of primary healthcare (PHC) and the health-related sustainable development goals (SDGs). PHC itself is an effective, efficient, and equitable approach for delivering essential health services to a significant proportion of the world’s population (WHO, 2019). From the perspective of the WHO, PHC provides a framework for the realisation of the 1978 Alma Ata Declaration of “health for all” (WHO, 1978). More recently, the SDGs re-emphasised the importance of PHC in addressing people’s health needs and preferences through comprehensive promotive, protective, preventive, curative, rehabilitative, and palliative care (WHO, 2019). To offer this mix of services to users, Ghana’s modern healthcare delivery system is integrated, multilevel, and spread all over the country. In this system, health facilities are either public, private not-for-profit, or private self-financing. The public health delivery system is decentralised, comprising of CHPSs as the first level to access healthcare, followed by sub-district health centres and poly clinics, district hospitals, regional hospitals, and teaching hospitals at the apex of the pile (See Figure 1).

Tertiary facilities are the teaching hospitals to which clients are referred from the primary and secondary level health facilities. These facilities provide specialist services and are staffed with specialists’ healthcare providers and consultants as well as general practitioners (GPs). Currently, there are five teaching hospitals in Ghana. These include Korle-Bu, Komfo Anokye, Tamale, Cape Coast, and Ho Teaching hospitals. However, there are concerns that this number falls short of the ratio of tertiary facilities needed to adequately provide quality health care to the over 30 million users in Ghana^1^. Secondly, having only one tertiary facility (Tamale Teaching Hospital) serving the population in the whole of the northern part of the country contradicts an important equity objective of the national health policy^2^. Secondary facilities are mainly the regional hospitals where doctors and other health professionals offer specialist care to clients who have been referred to them by staff at district/municipal hospitals or health centres who do not have the knowledge, resources, or specialisation to treat specific conditions. At the highest level of PHC delivery in the system are district or municipal hospitals. Staffed with GPs, physician assistants, midwives, nurses, and auxiliary staff, this category of health facilities serves as the primary sources of health care to some users, mostly those located near them. Other primary services that are beyond the expertise and capacities of the staff of health centres may be referred to the district or municipal hospital for further attention.

Further down the hierarchy of the health care delivery system are health centres. These facilities are located at the sub-district level and equipped to provide mainly PHC services that are not available at the CHPS centres. Thus, in addition to providing basic health care services, sub-district health centres serve as referral centres for services that are beyond the competences of staff at the CHPS. To effectively deliver this task, the Ghana Health Service recommends that these facilities are staffed at the minimum with a medical or physician assistant, a midwife, nurses, and auxiliary staff. However, it has been documented that a key challenge facing most of these facilities is limited availability of medical or physician assistants and midwives, particularly in rural areas (Domapielle et al., 2020). This results in frequent referral of clients from the sub-district level facilities to the district hospitals, a situation that has consequences for the quality of health care offered at that level. At the lowest level of the hierarchy of health care delivery are the CHPS. Led by a Community Health Officer (CHO), and supported by community volunteers, the CHPS programme sets the stage for the delivery of PHC by providing the first level of access to health care in the rural areas (Nyonator et al., 2005, Phillips et al., 2018). The primary focus of CHPS is to bridge the gap in access to basic quality health services in rural areas by strengthening community health services, and their main activities include the combination of improved nutrition, education on personal hygiene, family planning, vaccination services, treatment of common ailments, and injuries (Aikins et al., 2013). The staff also provide outreach services that are geared towards empowering rural women and vulnerable groups, and improving health provider, household, and community relationship. The CHPS concept represents a paradigm shift from a focus on curative care to an emphasis on preventing and controlling the spread of communicable and non-communicable diseases and promoting healthy lifestyles.

To strengthen this paradigm shift in the delivery of healthcare in the country, the Ministry of Health sought technical support from the government of Japan to expand the CHPS programme, which had been under implementation since 1999 (Aikins et al., 2013). In response to this request, the Japan International Cooperation Agency (JICA) implemented “The Project for the Scaling-up of CHPS implementation in the Upper West Region from March 2006 to March 2010. In this region, access to PHC services remains limited and health indicators are worse than they are in other regions in the country (Domapielle et al., 2021, Domapielle et al., 2020). The scaling-up of CHPS gave birth to FS (Aikins et al., 2013). FS is an approach to supervision that focuses attention on mentoring, joint problem-solving, and two-way communication between the supervisor and supervisee. In this approach, supervisors lead teams of health facilities’ staffs through a continuous process to better understand and meet clients’ needs. Facilitative supervisors at all levels do this by focusing on the needs of the staff (hereafter referred to as supervisees); they supervise and consider staff to be their own customers. This approach places emphasis on mentoring, provision of constructive feedback, joint problem solving, and dialogic communication between supervisors and supervisees (Frimpong et al., 2011, USAID, 2002, Aikins et al., 2013). The introduction of FS forms part of a paradigm shift from conventional monitoring to a new approach that incorporates participation in its genuine sense (Ofosu and Ntiamoah, 2016). In this regard, the supervisor will have the satisfaction of working as a team member, making supervisees learn and grow with improvement in quality of health, leading to staff motivation and commitment (USAID, 2002). Table 1 presents the guiding principles of FS.

**Table 1.** Guiding principles of facilitative supervision (FS)

*A client-oriented mindset*	Clients who visit the facility are considered external clients and staff of the facility are internal clients. Facilitative supervisors focus on the needs and expectations of both external and internal clients. For example, external clients have rights to quality health care, and staff have the resources necessary to deliver quality health care services. Facilitative supervisors keep these rights and needs in mind when assessing quality, involving staff to identify problems and find remedies to them.
*Staff involvement and ownership*	Supervisees are involved in the quality improvement process. Supervisors try to promote a spirit of ownership and teamwork by emphasising the importance and contribution of everyone in the provision of quality of health care services.
*Focus on processes and systems*	Facilitative supervisors emphasise the importance of improving processes and systems and not on mistakes made by individual staff. FS recognises that 75% of problems occur because of overly complex or faulty processes or systems.
*Cost consciousness and efficiency*	Poor quality is wasteful and costly, and good quality saves money. FS improves the quality and reduces cost both financially and in terms of the health of individuals and the community
*Continuous learning, development, and capacity building*	Facilitative supervisors pay close attention to staff development and capacity building. The approach involves the transfer of knowledge and skills needed to improve the quality of health care. Facilitative supervisors create opportunities for staff for training, refresher training, and training in new processes and procedures. They train staff to identify learning needs, develop plans on how to address these needs, and transfer the knowledge and skills acquired to other staff members.
*Ongoing quality improvement*	External supervisors visit facilities systematically to foster and sustain the quality improvement process. They continuously teach staff how to use different quality improvement tools and use them regularly. They also transfer the quality improvement tools to internal supervisors. Changes in the quality of services are regularly monitored and evaluated, while problem areas are constantly identified for improvement.

Adopted from USAID (2002:13)

## FS Process

FS visits are carried out quarterly by teams of health professionals in line with the hierarchical structure of the health delivery system. Thus, supervisors of district hospitals are drawn from the Regional Health Management Team, while supervisors of the sub-district health centres and CHPS are drawn from the District Health Management Team. At each level, a supervisory team would comprise a medical doctor, a physician assistant, a public health nurse, a midwife, a nutrition officer, a disease control officer, an M&E officer, a health information officer, an administrator, an accountant, and a storekeeper. In instances where funds are limited, the size of the supervisory team could be as small as four (4). These supervisors would have undergone comprehensive training on FS and have experience in the health care delivery system. Using the FS guidelines and checklists, supervisors assess, build relationships, and provide on-site training to supervisees (GHS, 2019). Each FS visit lasts 3–4 hours during which supervisory tools, including a supervision checklist, scorecards, observation, and interviewing are employed to assess the performance of supervisees.

Prior to FS visits, supervisors observe some mandated protocols. The first is the planning and preparatory stage, where the supervisory tasks are shared and movement plans drawn. Supervisors take into consideration the availability of human resources, funds, checklists, scorecards, reports assessment books, and communicate to the management of the facilities the date and time of the planned visits. The next step is the visit to the health facilities to perform the FS. At the facilities, the team would begin the process by holding a briefing session with the management, stating the purpose and approach to the FS. Supervisors’ interest at this stage is to ensure that supervisees actively involve themselves in all activities. The process progresses with a review of previous supervision reports to ensure that gaps identified during the previous supervision have been addressed. Following a review of the report, supervisory tools including checklists, observation, interviews, and scorecards are employed to assess supervisees performance. This process helps to identify progress as well as problems and challenges facing the facilities. The next stage of the process involves the provision of guidance, the identification of priorities and challenges, and developing action plans. Here, supervisors provide corrective measures by demonstrating the correct procedure to their supervisees and then task them to replicate it to confirm that they have understood the procedure. The team at this stage does not concentrate on individual supervisees but on the processes and the activities at the facilities. Following this, supervisees are given feedback on their performance; they are commended on areas they have done so well in, and the areas that need further attention are made known to them. It is also at this stage that consensus is reached on the action plan using a joint problem-solving approach. Documentation and reporting come as the fourth stage of the process where all the findings, activities as well as plans taken during the FS are put into writing and documented. Copies of the report are then given to the in-charges of the facilities. The fifth stage involves follow-up to provide continued support and keep supervisees in check. Monitoring and evaluation officers at the district and sub-district levels are presented with copies of the findings. This enables them to make a follow-up to check progress of work and to make sure that staff of facilities are adhering to standards and guidelines of the health care system. The final stage in the FS process is the review of report where monthly reports are revised and there is continuous communication with supervisors and supervisees to determine whether recommendations are being implemented.

Research has shown that FS helps in the reduction of routines and lower-level problems as staff learn to solve their problems with little technical assistance from higher-level supervisors (USAID, 2002, Frimpong et al., 2011, Aikins et al., 2013). However, research evidence on whether FS has contributed to improving the quality of PHC is limited. Previous studies in this field have only provided understanding on adherence to the tenets of FS. For instance, “Aikins et al. (2013) evaluation” of FS in the Upper West Region focussed mainly on adherence to some set benchmarks. It concluded that the adherence to the guidelines of FS in districts varied concerning the management of supplies, transport and equipment, information, meeting, and technical support. Additionally, Sumankuuro et al’s. (2018) discourse of Japanese Development Assistance and the Scaling-up of Community-based Health Planning and Services (CHPS) in Ghana mainly highlights the potential for FS to review and fine tune the administrative capacity of the relevant health administrators of the Upper West region with the primary objective to improve and enhance healthcare delivery. Furthermore, Bailey et al. (2016), in a systematic review of supportive supervision as a strategy to improve PHC services in Sub-Saharan Africa criticised the assumption that FS effectively builds capacity and enhances the quality of care provided by frontline health workers. They observed that although FS visits can increase job satisfaction and health worker motivation, the evidence on whether this translates to increased clinical competence and outcomes is mixed. This study bridges the existing knowledge gap by exploring the relationship between adherence to the tenets of FS and the quality of PHC services provision in a resource-poor context such as north-western Ghana. It is worth researching this relationship because one of the core objectives of the FS initiative is to improve the quality of PHC, which serves as an important determinant of facility visits and utilisation of health care services (Domapielle et al., 2020). The ensuing section focusses on the theoretical and conceptual frameworks of the study.

## Theoretical rationalisation of FS

McGregor’s 1960 theories X and Y provide a framework for exploring the rationale for implementing FS. The theory has two sides (X and Y) focussing on the inborn characteristics of the actors in the FS enterprise that will spur them onto participation. The X side of the theory states that human beings, by their nature, detest work, and engage in it as a matter of necessity. Advocates of the theory see supervision, monitoring and control, and motivation of employees as necessary conditions in ensuring that employees adhere to guidelines, rules, and regulations in the performance of tasks assigned to them. On the other hand, the Y side argues that employees characteristically love to work and that they have inner satisfaction in their career progression. The type of supervision advocated under Theory Y is that of facilitation, teaching, and mentoring. McGregor and his exponents are of the view that all supervisors are required to do is to provide a congenial, pleasant, healthy, and an engaging work environment for employees, who are highly motivated from within to implement activities for the delivery of expected outputs to achieve health services objectives and goals. Since the employees already have the organisation at heart, management needs to engage them in the decision-making process, create a friendly environment for mutual understanding and learning; and this will aid them unleash their skills and take responsibility for the success of the organisation. These will lead to the growth and development of the organisation and ensure capacity building and empowerment for both the health services supervisor and supervisee.

## Conceptual framework for assessing adherence to FS

**Figure 2.**
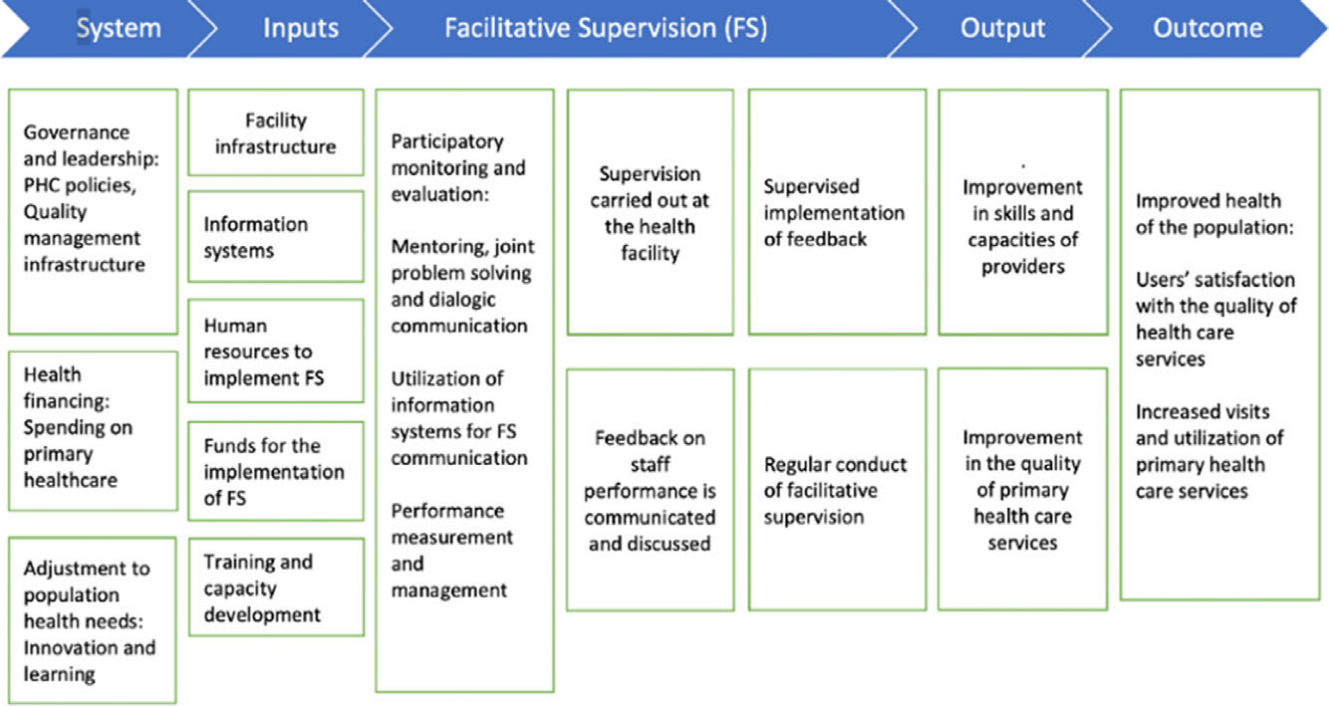
Conceptual framework for assessing adherence to facilitative supervision

Having rationalised the need to carry out FS, we adopt the Primary Health Care Performance Initiative (PHCPI) tool for measuring countries’ performance in PHC provision as the conceptual framework of the study. This framework is designed to support countries to identify priority PHC areas for improvement, track progress over time, and promote accountability for results (Ratcliffe et al., 2019, PHCPI, 2018a). For an FS programme to achieve its goal and objectives, it is important to examine the governance *system* under which it operates (Kweku et al., 2020, PHCPI, 2018a). Here, an analysis of FS as part of the PHC policy is critical in understanding whether for the objective of improving the quality of PHC, there has been a shift from conventional monitoring of health care provision, which is characterised by inspection and fault-finding assessment of health staff to a more participatory approach that incorporates joint problem-solving and continuous improvement in the quality of health care services provision (Ofosu and Ntiamoah, 2016). Analyses of the PHC policy would also provide an understanding of the change in the provision of mainly curative health services to the inclusion of and emphasis on preventive health services provision, public health issues, and reduction of taxes on health care services programme inputs (particularly the imported ones). Besides the policy considerations, the health needs of the population are also very worthy of consideration. Here, the concentration is on the doctor–patient ratios, nurse–patience ratios, facility–bed ratios, and the presence of certain facilities/equipment at various levels of health facilities. The next core element in the framework is *input.* For health care providers (supervisors and supervisees) to adequately execute the tasks assigned to them and achieve the objectives of FS, an analysis of the availability of essential health resources is critical. These resources would include human resources for health, health infrastructure (supervisors and supervisees), equipment, supplies and logistics, and funds. The availability of these inputs in their right quantities and quality sets the stage for the commencement of FS in the form of participatory monitoring and evaluation (PM&E), training and building capacity, and mentoring staff of health facilities (Kweku et al., 2020, PHCPI, 2018a, Ratcliffe et al., 2019). These activities will yield some immediate results in the form of *output, including* improvement in skills and capacities of health staff, improvement in the quality of PHC communication, discussion of feedback on staff, and improvement in communication. The final stage of the framework focuses on expected *outcomes*. It is expected that the successful implementation of FS will result in improved health of the population. In this regard, users’ satisfaction with the quality of health care services will increase and lead to increased uptake of PHC services (Kweku et al., 2020, PHCPI, 2018a, Ratcliffe et al., 2019). In a nutshell, this framework observes that since health service providers operate in a system, the proper functioning of all the FS programme characteristics will lead to the achievements of the goals and objectives of the FS. However, a dysfunction in one or more of these programme characteristics will result in failure. Figure 2 is the conceptual framework for assessing adherence to FS.

## Research context

The study was conducted in two districts, Wa Municipal and the Wa West district. The Wa Municipal has a total population of 200,672 people. From this figure, 98,493 are males, and 102,179 are females. Additionally, 143, 358 reside in urban areas and 57,314 reside in rural areas, making the area largely urban (GSS, 2021). It shares administrative boundaries with Nadowli District to the north, Wa East District to the east and to the west and the south, Wa West District. The municipality has Wa as its capital, which also serves as the regional capital of the Upper West Region (GSS, 2014a). In relation to the public sources of PHC, the municipality has 1 hospital, 10 sub-district health centres, and 47 functional CHPS zones (GHS, 2022).

**Figure 3.**
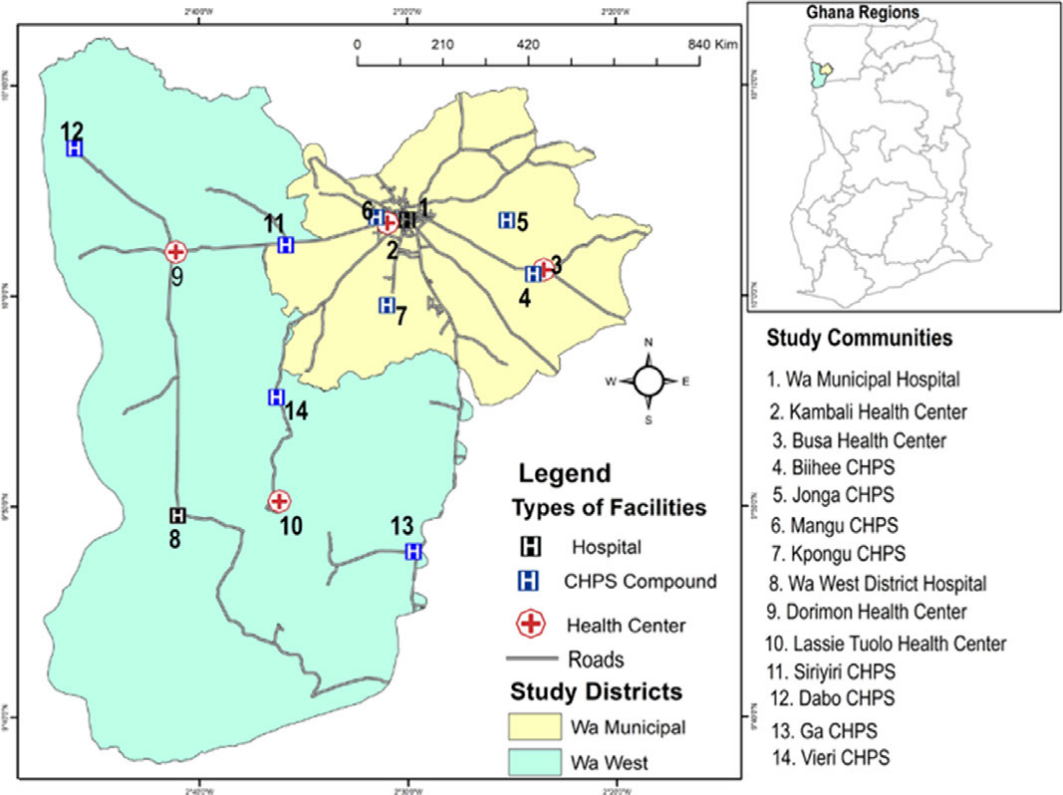
A context map of Wa municipality and Wa West district showing study facilities

The Wa West District was carved out of Wa District in 2004 by legislative instrument (LI 1751) under the Local Government Act 463, 1993. It has a total population of 96,957 people, of which 45,880 are males and 51,077 are females. Unlike the Wa municipal, all the communities in the Wa West district are described in the 2020 Population and Housing Census as rural and mostly poor (GSS, 2021). The district has Wechiau as its capital and shares boundaries to the south with the Savana region, to the north-west with Nadowli District, to the east with Wa Municipal and to the west with Burkina Faso (GSS, 2014b), [See Figure 3]. Compared to the Wa Municipality, the Wa-West District has 1 hospital, 8 sub-district health centres, and 40 functional CHPS zones (GHS, 2022).

**Figure 4.**
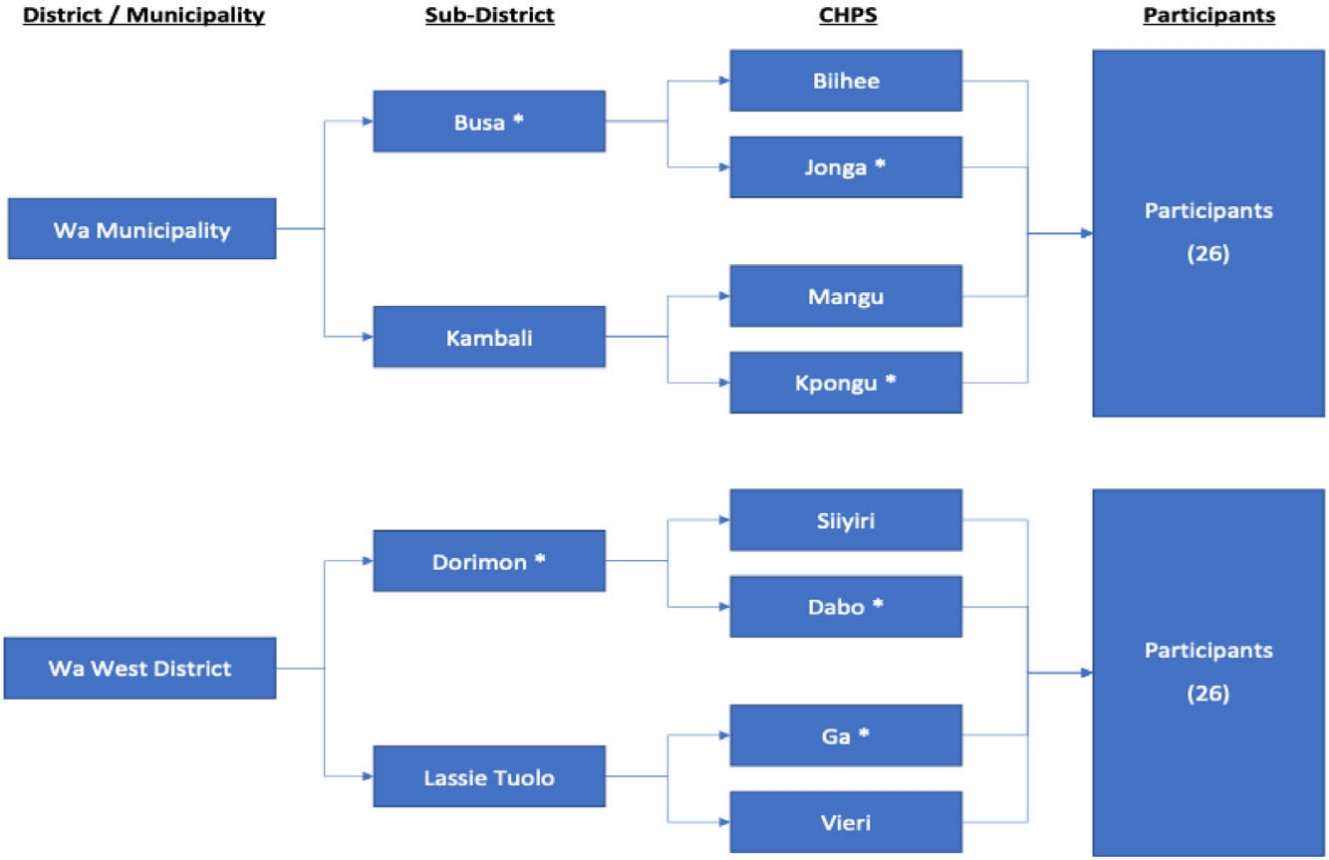
Multistage sampling approach employed in the study

Whereas the Upper West remains the poorest region in Ghana with a poverty rate of 70.7 per cent (GSS, 2018, GSS, 2015, GNHR, 2021), there is a wide variation in the headcount rate of poverty across districts, between the Wa Municipality (36%) and the Wa West District (90%) (GNHR, 2021). The selection of the Wa Municipality and the Wa West district for this study provides an appropriate setting for exploring whether the differences in resource endowment in these areas affect the extent of adherence to the tenets of FS and the quality of PHC services provision. Figure 2 is the context map of the Wa Municipality and the Wa West District.

### Research Design

A comparative exploratory design (multi-case study) was adopted to collect and analyse the data in the study. In addition to reviewing relevant literature on FS and uptake of PHC in Ghana and other developing contexts, we collected primary data through in-depth interviews and observation in the two districts. We also collected and analysed data drawn from relevant secondary sources, largely between 2015 and 2021.

### Sampling strategy

The multi-stage purposive sampling approach was used in the study. This was in recognition of the hierarchical structure of the health delivery system (Domapielle et al., 2021), which provides the framework for implementing FS. Thus, the first stage involved sampling of the two study districts; the second was the sampling of health facilities, and the third was the sampling of participants. In sampling the study districts, we considered the disparities in the distribution of health resources in favour of urban areas, and the accompanying variation in access to PHC among rural and urban populations in the region (Domapielle et al., 2020). Thus, we purposively sampled the most urbanised Municipality (Wa Municipal) and the most rural district (Wa West district), to enable us ascertain the differences and commonalities in adherence to the tenets of FS as well as the quality of PHC services provision in these contrasting locations. In the second stage, a total of 12 health facilities were purposively sampled; 6 in the Wa Municipality and 6 in the Wa West District. Distance from the district or municipal hospital was the criterion used in selecting the health centres. Again, our objective was to explore differences and commonalities in adherence to the tenets of FS as well as the quality of PHC services provision in health centres which are differentiated by distance to their respective higher health facility (hospital or health centre). In this regard, one health centre located less than 5 km from the district or municipal hospital, another located more than 5 km away from it, were purposively sampled. In Figure 4, sub-district health centres and CHPS centres that are located more than 5 km away from their respective higher-level facilities (hospital or health centre) are assigned an asterisk mark (*). Consistent with this approach, we sampled the Wa municipal hospital, two sub-district health centres (Busa and Kambali) and four CHPS (Biihee, Jonga, Mangu and Kpongu) in the Wa Municipality. Following the same hierarchical order, we also selected the Wa West District hospital, two sub-district health centres (Dorimon and Lassie Tuolo), and four CHPS (Siiyiri, Dabo, Ga, and Vieri) in the Wa West district. Figure 4 illustrates the multi-stage purposive sampling approach employed in the study.

The selection of study participants was in three stages. Stage one involved selecting 12 supervisors in total. These include two supervisors for each of the three levels in Wa Municipality and Wa West District (hospital, health centre, and CHPS). The participants were interviewed on-site immediately after the conduct of their supervisory routines. They provided in-depth information on the availability of resources and materials for carrying out FS, the extent to which supervisees adhered to the guidelines of FS, the differences, and commonalities in the level of adherence between facilities located in urban areas and those located in rural areas, and whether the extent of adherence to the guidelines of FS had influenced the uptake of PHC services at the various health facilities. The second stage involved the selection of 20 supervisees: 2 each from the two selected hospitals, 2 each from the selected sub-district health centres, and 2 each from the selected CHPS facilities. Like the supervisors, these supervisees were purposively selected and interviewed on the regularity of FS at their facilities, the availability of resources and materials for FS, their willingness and ability to adhere to the guidelines of FS, and the effect of FS on the uptake of PHC services. The third stage involved the selection of users of PHC services through convenience sampling. The inclusion criterion was users who had used the same primary health facilities prior to the implementation of FS such that they would be able to compare the quality of health care provision then and now. In all, 20 users of PHC services were sampled; 2 from each of the health facilities studied. They were interviewed for their views on the quality of health care since the introduction of FS in their primary health facilities. The responses of all these study participants made it possible to critically analyse the extent to which FS has contributed to the quality and uptake of PHC services in the region.

### Data collection

In-depth interviews and non-participant observation were used to collect data for the study. With the aid of semi-structured interview guides, in-depth interviews were conducted with supervisors, supervisees, and users of health care services in the two districts. Interviews were conducted in English for interviewees who spoke it and in Dagaare (the local language) for users who do not speak it. Interviews were audio-recorded for transcription, coding, and analysis.

Non-participant observation was also carried out to compliment data gathered via interviews. Using an observation checklist at the various levels of FS, we observed and recorded evidence of the availability and use of supervisory materials such as visits book, job aids, score cards etc. for FS. We also observed for evidence of coaching and mentorship in the performance of FS, and for evidence of supervisees’ willingness to adhere to the guidelines of FS in the provision of PHC to clients, for the amount of time spent.

### Data processing and analysis

Following the data collection, a thematic analytical framework was used to analyse the relationship between adherence to the principles of FS and uptake of PHC services in the study municipality and district. Thus, the analytical process started right from the stage of developing the data collection instruments, where the questions were structured to focus on the objective of the study. The process involved transcribing and getting familiar with the data. It also involved reading each interview transcript line by line, noting down repetitions, similarities, and differences relevant to the research questions. In the margins of each page, we wrote down the main themes from the page’s conversation. From this preliminary analysis, we examined the themes a second time and then put them into the thematic networks. For the final phase, we used the soft copies of the transcripts to pull together the segments of data that represented each theme and developed qualitative analysis by analysing in detail what supervisors, supervisees, and users said about these themes and what they signified in relation to the research question. In terms of secondary analysis of data, the study relied significantly on the annual reports of the Ghana Health Service and the Ministry of Health.

### Ethical Issues

Ethical issues involving research with human participants, including anonymity, confidentiality, and consent, were considered at the outset of the research. Following the selection of the Wa municipality and the Wa West district, permission was obtained from the Upper West Regional Health Directorate to carry out the study. Approval was also obtained from the Wa Municipal Health Directorate and the Wa West District Health Directorate to carry out the study in their respective administrative areas. Informed consent was sought and obtained from all participants before conducting each interview. Participants were made aware that their decision to participate in the study was completely voluntary. They were free to withdraw from the study at any time, and they could skip any question(s) they did not wish to answer. All information provided by participants was treated as strictly confidential.

## Results

The results compare the practice of FS in the Wa West district and the Wa Municipality on the bases of regularity of supervision, adherence to the guidelines of FS, and the effect of FS on uptake of PHC. Important interview extracts are presented in this section to add credibilty to the results.

### Regularity of FS

In relation to the regularity of FS at health facilities the results show that neither the Wa Municipality nor the Wa West district managed to carry out the mandated quarterly supervisory visits to health facilities. Participants attributed this to limited availability of resources including funds. A supervisor at the Wa Municipal Hospital disclosed that:

*“FS has recently not been as consistent in all the districts as it was in the past because of limited funds. We hardly conduct all planned FS visits in a year. We carry out two or three instead of four supervisions in a year.”*

Similarly, a supervisor at the Lassie Tuolo Health Centre in the Wa West District expressed disappointment in the inability of the District Health Directorate to provide funds for regular FS visits. This is what she had to say:

*“When JICA [NGO] was funding the implementation of FS we did not miss any visits because the funds were always available to procure the supervisory materials and logistics and pay travel expenses. Since their departure the District Health Directorate has been struggling to raise funds for us conduct the required quarterly visits.”*

It is worthy of note, however, that while both districts were usually unable to carry out all their quarterly supervisions, Wa Municipal tended to carry out more supervisions than the Wa West. We found that supervisees at health facilities in the Wa Municipality received relatively more supervision than their counterparts in the Wa-West district. For example, at the beginning of the third quarter of 2020, the supervisees at the Wa Municipal Hospital had received two supervisory visits. A supervisee at the hospital was impressed with the regularity of FS:

*“Supervision has been consistent in this facility for the past three years. Supervisors visit this facility once every four months within a year. And so, in a year we have like three facilitative supervisions. We are, however, yet to be supervised during this third quarter. I am sure that supervisors will be here to do the supervision”*

In contrast, their counterparts at the Wa West district hospital had been supervised only once for the same period. A supervisee at the Wa West district hospital revealed that:

*“Only two out of the scheduled four supervisions were carried out in the whole of 2018. The same schedule was repeated in 2019, and as of July 2020, only one supervisory visit had been carried out at the facility.”*

Similar disparities in FS visits were observed at the sub-district health centres as health facilities in the Wa Municipality recorded more supervisory visits than health facilities in the Wa West district. For example, whereas three supervisory visits were conducted in the Kambali sub-district health centre in the Wa Municipality in 2019, only two of such visits were made to the Dorimon sub-district health centre in the Wa West district in the same year. Similarly, whereas two supervisory visits were recorded for the Mangu CHPS in the Wa Municipality in 2019, only one visit was paid to the Dabo CHPS in the Wa West district in the same year. A supervisee at the Dabo CHPS had this to say about the regularity of supervisory visits to the facility:

*“The supervision sessions are so helpful, and we learn a lot of new ways of improving the quality of services we provide. Unfortunately however, they [supervisors] come here only once a year with the explanation that the funds for supervision are limited.”*

These results demonstrate that, in general, both the Wa Municipality and Wa West district did not achieve the mandated quarterly FS visits to health facilities. However, the Wa Municipality tends to carry out more supervisory visits at all the levels of health care than the Wa West district. The main reason for this disparity is the limited availability of funds, and the Wa West District is poorer (GSS, 2021).

### Adherence to the guidelines of FS

In addition to the regularity of FS, we also explored the extent to which staff of facilities adhere to the guidelines of FS and found that, unlike the regularity of FS where disparities occurred, adherence to the FS implementation guidelines was generally good in both districts. Supervisors were impressed that although supervisees are not forced to follow guidelines, they adhere to them to a great extent. They commended them for occasionally improvising in the absence of essential supervision materials such as referral forms, standardised notebooks etc. In the same way, supervisees from both the Wa Municipality and Wa West district corroborated that even when essential items like a standardised attendance book were not available, they would often improvise with a notebook to record attendance. We observed however, that improvisation was common only at the health centres and CHPS. The two hospitals studied did not report this practise. A supervisee at the Kpogu CHPS in the Wa Municipality said this about adherence to the guidelines of FS:

*“We do follow the guidelines to a greater extent without being pressured or forced to. In some situations, we improvise to enable us to follow the guidelines. For example, we have a standardised notebook that we use for recording the attendance of clients. However, whenever we are short of the notebook, we buy an ordinary notebook book, rule the lines, and use it for the same purpose.”*

At the Lassie Tuolo Health Centre in the Wa West District, a supervisor corroborated the above statement saying:

*“I am impressed about their [supervisees] willingness to follow the guidelines prescribed to them. I have observed that even when some essential tools and materials are missing, they would improvise and get the work done appropriately.”*

Similarly, a supervisee at the Kambali Health Centre said this about improvising to adhere to the guidelines of FS:

*“It is always our wish to adhere to these guidelines to the letter. The problem here however is that we do not have the required resources to work with. For example, last week we bought a notebook to use as a register because the official register is full. We cannot say we would not keep records of clients of our facility because there isn’t a register and so we have to improvise”*

Despite efforts to adhere to the guidelines through improvisation, supervisees in remote rural health centres and CHPS bemoaned that some improvised measures were risky. Some supervisees recalled the several instances when they have carried clients on motorbikes to the regional hospital for further treatment because there is no ambulance service in the district. At Wa West District Hospital, a supervisee bemoaned that:

*“Severally, we have taken risks by resorting to using of motorbikes to transport referred clients to the Wa regional hospital because there is no ambulance in the district.”*

The risk of transporting referred clients on motorbikes was not limited to the Wa West District. We found that sub-district health centres in distant communities in the Wa Municipality also transport clients using motorbikes. Supervisees at the Busa Health Centre had this to say about the improvised use of motorbikes and tricycles to transport clients to the municipal or regional hospital:

*“We do our best to refer promptly to a higher-level facility and because of the difficulty in securing ambulance services the male nurses here would often use our motorbikes to carry the patient to the Municipal hospital for further treatment.”*

Thus, whereas supervisees’ willingness to adhere to the tenets of FS is unquestioned, the constraint of limited resources remains an impediment to the smooth delivery of health care, particularly in remote rural areas.

### Contribution of FS to the Quality of Primary Health Care

This section presents results on the relationship between FS and improved quality of PHC in the study districts. In this regard, supervisees from both the Wa Municipality and Wa West district concurred that the implementation of FS has improved their professional skills and the quality of health care delivery at the various health facilities. In terms of professional skills, the study revealed that both supervisors and supervisees have become more disciplined, enabling them to stick to prescribed guidelines in providing quality health care. A sub-district supervisor with whom we interacted at the Vieri CHPS said this about improvement in the level of discipline ushered in by the training she has received through FS:

*“We go strictly by the guidelines when supervising, and I have observed that the staff I supervise at the CHPS have become very disciplined too. This has reduced the errors and mistakes that had characterised the provision of health care at the lower level.”*

Similarly, a Supervisee at the Biihee CHPS commented on the effect of FS visits on the quality of health care being delivered in the facility:

*“The coaching and mentoring and the availability of standard treatment guidelines has given me confidence to provide treatments to clients without the fear of making the mistakes I made in the past. I have learned a lot from my supervisors through these FS visits and the clients who visit the facility express appreciation for the quality of care they receive.”*

In support of this observation, supervisees reported that the excellent relationship with their supervisors had built in them the ability to relate warmly and communicate effectively with clients. A supervisee at the Ga CHPS offered this explanation in connection with improved communication with clients:

*“Communication is key in every field, especially for those of us in the health sector; hence good interpersonal relationships and your ability to communicate well to clients who visit your facility is a must, and the application of FS has helped in this direction. In addition, the good interpersonal communication skills we have imbibed through the supervision process have contributed significantly to our ability to interact cordially with clients.”*

The foregoing statements are corroborated by our observation of some of the FS sessions at the health facilities. We observed during these sessions at the hospitals, Health centres, and CHPS that supervisees used hard copies of the standard treatment guidelines and provided services with confidence. We observed again that supervisees communicated very well with clients; they would warmly greet and welcome them when they arrive at the facility, offer them a seat, and explain the procedure to them.

In terms of the extent to which FS has contributed to the overall quality of health care, supervisors at all three levels of health care provision reported that because of the implementation of FS, supervisees now understand why data are collected and how to use data in the provision of health care. Additionally, FS has built the capacities of health staff to effectively allocate both human and logistical resources, which has contributed towards improving PHC delivery. At the Kambali Health centre in the Wa Municipality, a supervisee remarked that:

*“Facilitative supervision has helped us to correct so many things we were doing wrong. In the past we neither planned activities nor allocated resources based on any specified criteria. Today, all activities are planned; implementation is based on the plan, and resources are allocated based on a specified criterion.”*

We also uncovered through the interviews with participants and from the district health reports that attendance for maternal health care services had increased following the implementation of FS. For example, there was consensus among participants from the two districts that prior to implementing FS, maternal and child health care attendance was not encouraging. However, following the inception of FS, facilities have recorded high attendance. The following quote sums up the consensus expressed by supervisees interviewed:

*“Before the start of FS, we used to have very few pregnant women visiting this facility, very few nursing mothers bringing their babies for Routine Immunization (RI) and a few people coming for family planning. It became a worrying situation for us. During one of the FS sessions, staff decided to put the issue before our supervisors as one of the facility’s problems. It was then that our supervisors took us through how to communicate and relate well with clients. After that session, we started implementing what we were taught, and it worked perfectly well. We began recording increases in attendance of clients”.*

Our review of CHPS facilities’ records confirmed increases in antenatal visits a year following the implementation of FS. Table 2 provides evidence of increases in antenatal visits to CHPS facilities in the Wa Municipality and the Wa West District in 2007.

**Table 2.** Antenatal visits to CHPS facilities in the Wa Municipality and the Wa West District in 2007.

District/Municipality	CHPS	Percentage increase (%)
Wa Municipality	Biihee	17
	Jonga	13
	Mangu	10
	Kporngu	11
Wa West District	Siiriyiri	17
	Dabo	10
	Ga	22
	Vieri	15

Additionally, supervisors and supervisees attributed improved patient satisfaction to the implementation of FS. Overall, supervisors and supervisees acknowledge that the intervention has contributed significantly towards improving the delivery of quality PHC in the districts studied.

## Discussion

This comparative study unearthed important findings which have implications for health policy design and implementation in Ghana and beyond. Firstly, we found that variation in the regularity of FS in the two districts did not affect the extent of adherence to tenets of FS in health facilities. We also uncovered that the implementation of FS positively affected the quality of health care delivery in the districts. The latter is novel and calls for further research to ascertain the possibility of a correlation between improved quality of health care occasioned by FS and increased uptake of PHC services in the country and beyond. These findings are discussed in detail in the subsequent thematic sections of the article.

Regularity of supervision is a prerequisite for ensuring that the goal and objectives for implementing FS are achieved. Two interesting findings emerged concerning the regularity FS in the two districts. The first is that both districts failed to achieve the quarterly supervision of health care provision in health facilities as spelt out in the FS guide. Second, the health facilities in the Wa Municipality received more supervision sessions than those in the Wa West district. Whereas health facilities in the Wa Municipality received up to three supervisory visits per annum instead of the required four visits, the maximum number of supervisory visits received by any health facility in the Wa West district was two. The main reason attributed to this inadequacy is the limited availability of health resources ranging from funds, logistics, and supervisors. While limited resources for health remains a challenge facing both districts, the situation in the Wa West district was found to be worse for two main reasons. The first reason is the pervasiveness of poverty in the district (GSS, 2021, GSS, 2015), which affects its capacity to generate internal revenue to finance the cost of implementing FS activities. Secondly, the district’s poverty situation is compounded by its large size and dispersed settlement pattern^3^. In this regard, long travel distances from Wechau^4^ to sub-district health centres and CHPS increase the costs of conducting FS, and thus limits the number of supervision sessions that are fiscally affordable. This finding is consistent with previous research findings where constraints in the conduct of PHC provision’s supervision were attributed to limited availability inputs (Aikins et al., 2013, Kweku et al., 2020, Ratcliffe et al., 2019). A related finding that is of interest to this research is the revelation that the variation in the regularity of supervision in the districts did not create discrepancies in supervisees’ level of adherence to the guidelines of FS. Although this finding was reported earlier by Aikins et al. (2013) in their study of FS in PHC service delivery in Northern Ghana, it is important to discuss it within the framework of McGregor’s (1960) X and Y theory. Whereas the finding contradicts the X side of the theory as well as the lineal input-output relationship of the Primary Health Care Performance Initiative (PHCPI) framework for implementing FS (PHCPI, 2018a, Ratcliffe et al., 2019), on the contrary, the finding can be associated with the Y side of McGregor’s theory, which espouses that employees characteristically love to work and that they have inner satisfaction in accomplishing tasks, which in their view, contributes to career progression (McGregor, 1960). Evidence of adherence to the guidelines despite limited supervision demonstrates a high sense of commitment on the part of the supervisees in the Wa West district to provide quality health care to the population.

Most significantly, the study has established a relationship between FS and the quality of PHC in the two districts. We found in both districts that adherence to the guidelines of FS had resulted in improvement in the quality of PHC. The implementation of FS improved the skills of providers in the collection, analysis, and utilisation of data in the provision of PHC. Providers concurred that the intervention has built their capacity to communicate appropriately with clients and relate with them in ways that are deemed client-friendly and appropriate. Appropriation of health resources also emerged as one of the positive effects of the intervention on the quality of PHC. In this connection, providers have seen improvement in their competence and ability to efficiently allocate health personnel and logistics in the provision of PHC. Participants observed that improvement in the quality of health care, triggered by FS is reflected by a remarkable reduction in errors committed by providers, the confidence of providers in the administration of care and the satisfaction of PHC users with quality of health care they have received recently. This finding is important both for research and policy purposes. In terms of research, it concurs with the horizontal conceptualisation and implementation of FS in PHC which includes the readiness of the health system, availability of inputs, effective implementation of FS, resulting in expected outputs and outcomes (PHCPI, 2018b, PHCPI, 2018a, Ratcliffe et al., 2019). On the backdrop of this finding, further research is needed to establish whether improvement in the quality of PHC has resulted in increased facility visits and utilisation.

From a policy perspective, the finding reflects the relevance of the PHCPI tool for measuring countries’ performance in PHC provision. It illuminates and reinforces the need for innovation in health resources mobilisation accompanied by targeted investment in FS. By providing such a specific policy attention to FS, the challenges of limited human and logistical resources will be tackled; supervision will be regular, materials will be available, and adherence to the guidelines will be deepened to sustain improvement in the quality of PHC delivery in underserved areas.

## Conclusion and implications for planning

In this study, we explored the correlation between the implementation of FS and the quality of PHC services in two contrasting districts in north-western Ghana. We found that health facilities in the Wa West district were relatively underserved both in human and logistical resources, reducing the regularity of their supervisory visits compared to the Wa Municipality. This notwithstanding, adherence to the prescriptions of FS was rated as satisfactory in both districts, culminating in improvement in the quality of PHC. To promote and sustain the regularity of FS for improved quality of PHC delivery, health planners, and implementers should focus on addressing the challenges of limited logistics and inadequate human resources for health. This would require innovation in the mobilisation of resources accompanied by targeted investment in FS, distribution, and monitoring of the use of PHC resources. Based on the findings, we advocate further research to establish whether improvement in the quality of PHC has resulted in increased facility visits, utilisation of health care services and progress in the overall health of the population in the region.

### Limitations of the study

The study covered a limited number of health facilities and therefore the findings are not meant to be generalised to reflect the contribution of FS to improved PHC in Ghana. This notwithstanding, the findings are indicative of what pertains in the north-western part, which suggests that similar results could be obtained in other parts of the country and beyond by replicating the same methodological approach.

## Footnotes

1 GSS 2021. 2020 Population and Housing Census Report. Accra: Ghana Statistical Service.

2 Policy Objective 1: To strengthen the healthcare delivery system to be resilient by Ensure equitable access to the appropriate quality and quantity of health workers as well as the availability and appropriate use of quality medicines and medical products.

3 Fig. 2 shows a map of Wa municipality and Wa West district which highlights among others the large size and dispersed settlement pattern of the Wa West district in contrast to the Wa Municipality.

4 Wechau is the district capital of the Wa West district in the Upper West Region of Ghana.
